# Enhanced lipid and biomass production by a newly isolated and identified marine microalga

**DOI:** 10.1186/s12944-016-0375-4

**Published:** 2016-12-05

**Authors:** Mouna Dammak, Sandra Mareike Haase, Ramzi Miladi, Faten Ben Amor, Mohamed Barkallah, David Gosset, Chantal Pichon, Bernhard Huchzermeyer, Imen Fendri, Michel Denis, Slim Abdelkafi

**Affiliations:** 1 Biotechnologie des Algues, Biological Engineering Department, National School of Engineers of Sfax, University of Sfax, Sfax, Tunisia; 2 Institute of Horticultural Production Systems, Section Biosystems Engineering, Leibniz University Hannover, Herrenhauser Str. 2, 30419 Hannover, Germany; 3 Center for Molecular Biophysics (CBM), CNRS UPR4301, Orléans, France; 4 Institute of Botany, Leibniz Universitaet Hannover, Herrenhauser Str. 2, 30419 Hannover, Germany; 5 Unit Research of Toxicology-Microbiology Environmental and Health UR11ES70, Faculty of Sciences of Sfax, University of Sfax, Sfax, Tunisia; 6 Aix Marseille Université, Université de Toulon, CNRS/INSU, IRD, Institut Méditerranéen d’Océanologie (MIO), 163 avenue de Luminy, Case 901, 13288 Marseille Cedex 09, France

**Keywords:** Microalgae, *Tetraselmis* sp., Response surface methodology, Lipids, Flow cytometry, Poly-unsaturated fatty acids

## Abstract

**Background:**

The increasing demand for microalgae lipids as an alternative to fish has encouraged researchers to explore oleaginous microalgae for food uses. In this context, optimization of growth and lipid production by the marine oleaginous V_2_-strain-microalgae is of great interest as it contains large amounts of mono-unsaturated (MUFAs) and poly-unsaturated fatty acids (PUFAs).

**Methods:**

In this study, the isolated V_2_ strain was identified based on 23S rRNA gene. Growth and lipid production conditions were optimized by using the response surface methodology in order to maximize its cell growth and lipid content that was quantified by both flow cytometry and the gravimetric method. The intracellular lipid bodies were detected after staining with Nile red by epifluorescence microscopy. The fatty acid profile of optimal culture conditions was determined by gas chromatography coupled to a flame ionization detector.

**Results:**

The phenotypic and phylogenetic analyses showed that the strain V_2_ was affiliated to *Tetraselmis* genus. The marine microalga is known as an interesting oleaginous species according to its high lipid production and its fatty acid composition. The optimization process showed that maximum cell abundance was achieved under the following conditions: pH: 7, salinity: 30 and photosynthetic light intensity (PAR): 133 μmol photons.m^−2^.s^−1^. In addition, the highest lipid content (49 ± 2.1% dry weight) was obtained at pH: 7, salinity: 37.23 and photosynthetic light intensity (PAR): 188 μmol photons.m^−2^.s^−1^. The fatty acid profile revealed the presence of 39.2% and 16.1% of total fatty acids of mono-unsaturated fatty acids (MUFAs) and poly-unsaturated fatty acids (PUFAs), respectively. *Omega 3* (*ω3*), *omega 6* (*ω6*) and *omega 9* (*ω9*) represented 5.28%, 8.12% and 32.8% of total fatty acids, respectively.

**Conclusions:**

This study showed the successful optimization of salinity, light intensity and pH for highest growth, lipid production and a good fatty acid composition, making strain V_2_ highly suitable for food and nutraceutical applications.

## Background

Marine photosynthetic microalgae are potential producers of various bioactive substances such as vitamins [1], pigments [2, 3], poly-unsaturated fatty acids (PUFAs) [2, 4], triglycerides [5] and polysaccharides [6]. In fact, the marine oleaginous microalgae have been used in food and nutraceutical applications [7, 8] as a great source and producers of good lipids and PUFAs such as *omega 3* (EPA (C20:5), DHA (C22:6), α-Linolenic (C18:3 (n-3))), *omega 6* (C18:2), γ-Linolenic (C18:3 (n-6)) which are very important for human health and treatment of disease such as cancer, Alzheimer’s, modulatory vascular resistance, atherosclerosis and infant malnutrition [9]. Microalgae species generate some natural adaptation mechanisms under several, even noxious, culture conditions. These mechanisms induced modifications in their biochemical composition, like changing intracellular fatty acid biosynthesis as a protection against osmotic stress resulting from salinity changes [10]. Many researches conducted on lipid metabolism showed that several factors could affect lipid biosynthesis and their accumulation in microalgae, such as high light intensity [11–13], high salinity [14, 15], nitrogen and phosphorus starvation [16, 17], temperature [18–20] and pH [21]. In fact, light intensity and salinity are major environmental factors that affect photosynthesis and enzymatic activities. Some findings demonstrated that tuning light intensity alone could increase lipid content in green microalgae. Evidence was also reported for lipid production increase at low pH (6) [21]. However, the combined effects of environmental factors on lipid biosynthesis by green microalgae remained poorly documented. Microalgae cells have to reduce free radical synthesis under stressful culture-conditions by inhibiting electron accumulation in thylacoid membranes [22]. Under high-light-intensity stress, the induction of the enzyme pathway for carbon fixation was associated with a high electron flux [11]. Consequently, carbon fixation resulted in producing a triose phosphate as a primary product that can be involved in lipid or starch biosynthesis [22]. Salinity stress can lead to decrease or stop microalgal growth, biomass production and conversion of photosynthetic energy to chemical energy for fatty acid and starch synthesis [10]. According to Rodolfi et al. [23] and Studt [24], green microalgae cultures can produce oil with a yield 5 to 20 times that of common plant under stress culture conditions [25, 26].

Among 30 000 strains that have been isolated and identified [27], the green marine microalgae *Tetraselmi*s sp. (Chlorophyta) was selected as a model strain that is able to grow under several culture conditions due to the following criteria: its photosynthetic pathway versatility, its important nutritional value (lipids, pigments, etc.) and lack of toxicity [28, 29]. It was reported by Mata et al. [30] that *Tetraselmis* sp. was the most known microalgae that produce high lipid content. In fact, lipid composition of *Tetraselmis* sp. was strongly modified by culture conditions [11, 31]. Thus, this species is considered to be an essential source of PUFAs, especially eicosapentaenoic acid (EPA) [32]. According to Huang et al. [33], total lipid content of microalgae *Tetraselmis subcordiformis* reached up 33.72% of dry weight (DW) when it was cultivated in presence of 1.2 mM ferric ion. Marine green microalgae species such as *Nannochloropsis* sp. produced a total lipid content of more than 47% DW when cultivated at optimal temperature, salinity and light intensity [10]. In this study, the marine green microalga *Tetraselmis* sp. suitable to lipid production was isolated and identified based on 23S rRNA gene. Response surface methodology (RSM) coupled to Box-Behnken design (BBD) was applied to optimize responses and to analyse the effect of environmental factors and their interactions. The enhancement of lipid content upon tuning environmental conditions was monitored by gravimetric method and flow cytometry (FCM) after staining cells with Nile red (NR), a lipophilic fluorescent probe NR [34, 35] and the lipid bodies were observed by epifluorescence microscopy. To our knowledge, the analysis of lipid accumulation by flow cytometry (FCM) after staining with NR in *Tetraselmis* species isolated from the Mediterranean Sea was not reported up to now. Statistical methods were applied to develop low cost culture systems and to achieve maximum cells abundance and lipid production by this strain.

## Methods

### Isolation, purification and molecular identification of microalgal strains

#### Enriched cultures

Seawater samples were collected at the Tunisian Coast of Sidi Mansour (Mediterranean Sea). Samples (200 cm^3^) were first passed through a 60 μm pore size membrane to remove protozoa. Then, the filtered seawater was passed successively through 3 membranes with 20.0, 0.4 and 0.22 μm pore size, respectively. Thereafter, 2 cm^3^ of the final filtrate were transferred into sterile tubes and each membrane was immersed into a 250 cm^3^ flask containing 50 cm^3^ of F/2 Provasoli medium [36]. Every 3 days, the flasks and tubes were examined and the algal growth was monitored by inverted microscopy (Motic microscope AE2000, Spain) at 40× magnification.

#### Isolation of microalgae

After initial cultivation of the samples with F/2 medium, pure cultures were isolated by performing serial dilutions, plating and by the use of micromanipulation methods. Individual colonies were routinely maintained on both liquid and agar slants of F/2 medium by regular sub-culturing for 15 days and regularly examined by microscopy.

#### Cultivation

Cultures of V_2_ strain were maintained in 250 cm^3^Erlenmeyer flasks containing 150 cm^3^ seawater complemented with F/2 medium (pH 7) at 25 °C and continuously illuminated at a photosynthetic light intensity (PAR) of approximately 84 μmol photons.m^−2^.s^−1^ (TL5 tungsten filament lamps; Philips Co., Taipei, Taiwan), except for the medium and high (133 and 182 μmol photons.m^−2^.s^−1^, respectively) irradiance experiments. All the cultures were initiated by supplementing the F/2 medium with 10% (v/v) inoculum (concentration of algal stock cultures) and incubated for 15 days.

### DNA isolation, sequencing and phylogenetic analysis

Genomic DNA was extracted by a chloroform/isopropanol method from 10 cm^3^ of algae suspension that was sedimented by centrifugation at 4500 × *g* for 10 min. The algal suspension was collected at the end of the exponential phase of the related culture [2] as described in Haase et al. [37]. Two primers, namely, p23SrV_f1 (5′GGA CAG AAAGAC CCT ATG AA 3′) and p23SrV_r1 (5′ TCA GCCTGT TAT CCC TAG AG 3′) were used for Phusion PCR reaction [37]. The 23S region [38] of the DNA gene sequence was determined by Seqlab, Germany and the blast from the National Centre for Biotechnology Information (NCBI) was used to compare nucleotide sequences homologous to the genes sequenced from the studied eukaryotic species. Multiple nucleotide sequence alignments were achieved by using CLUSTALW [39]. The phylogenetic tree was constructed using the Neighbour-Joining method boot strapping [40]. The statistical significance of the resulting dendrograms was calculated by bootstrapping on 1000 replicates and the values of bootstrap were shown as percentages.

#### Growth measurement

The microalgae growth was determined by estimating cell concentration from the absorbance at a 680 nm (i.e., A_680_) measured with a spectrometer (T60-UV-Visible Spectrometer, UK). After cultivation, cell pellets were obtained by centrifugation at 4500 × *g* for 10 min at late exponential phase. Pellets were then dried at 105 °C until their weight kept constant. The dried microalgae were weighed to determine their dry biomass weight (DW). Data of growth in Box-Behnken experiments were presented as mean with standard deviation (±SD).

#### Total lipid extraction

Total lipid extraction was carried out from dry biomass according to the method of Folch et al. [41] as modified by Bligh and Dyer [42]. The dry cells from 50 cm^3^ cultures were extracted using 3 cm^3^ chloroform/methanol/water (2/1/1). Then, the mixture was agitated for 15 min in orbital shaker at 100 rpm at room temperature. The extract was centrifuged (10 min at 8000 × *g*) and the organic phase was recovered. The pellet was re-extracted in 3 cm^3^ chloroform/methanol/water solution three times. Finally, the solvent phases were combined and evaporated to yield the lipid content that was calculated using the following equation: Lipid content(%) = W_L_/W_A_ × 100%

Where W_L_ (g) is the extracted lipids weight and W_A_ (g) is the dry algae biomass.

Gravimetric analysis of lipid content of Box-Behnken experiments were performed in duplicate, and data were presented as means with standard deviation (±SD).

### Experimental design and analysis of the response surface

The level of the significant factors and the interaction effects between culture conditions which influence cells abundance and lipid production were analyzed and optimized by Box-Behnken methodology [43]. In this study, the experimental design contained 15 trials for optimizing culture conditions and the independent variables were studied at three levels, which are low (−1), medium (0) and high (+1) (Table 1) [44].

**Table 1:** Variables and experimental levels for optimising culture conditions

Factors	Coded symbol	Levels		
Levels		−1	0	+1
Salinity	X_1_	20	30	40
Light intensity (μmol photons.m^−2^.s^−1^)	X_2_	84	133	182
pH	X_3_	6	7	8

The growth (Y_1_) and lipid content (Y_2_) were taken as the response values of the design. The different factor levels and response values are shown in Table 1 and Table 2, respectively. The optimal values were derived from the result analysis by using the NemrowdW Software (LPRAI, Marseille) [45]. The experimental data obtained from the Box-Behnken model experiments can be represented by the following quadratic polynomial Eq. (1):

**Table 2:** Results from Box-Behnken experiments: optimizing culture conditions for V_2_ strain growth and lipid production

Exp no.	Salinity (X_1_)	Light intensity (X_3_) (μmol photons. m^−2^.s^−1^)	pH (X_3_)	Growth (A_680 nm_) (Y_1_)	Lipids (a.u.) (Y_2_)
1	30	84	6	0.54 ± 0.013	1836.05 ± 0.08
2	40	133	6	0.56 ± 0.0205	2031.44 ± 0.285
3	30	182	6	0.57 ± 0.0305	2198.52 ± 0.075
4	20	133	6	0.57 ± 0.012	1103.61 ± 0.012
5	20	84	7	0.44 ± 0.016	978.00 ± 0.26
6	40	84	7	0.51 ± 0.033	1491.41 ± 0.21
7	40	182	7	0.52 ± 0.022	2675.11 ± 0.2
8	20	182	7	0.60 ± 0.001	980.76 ± 0.06
9	30	84	8	0.45 ± 0.0015	1860.83 ± 0.32
10	20	133	8	0.57 ± 0.02	1400.50 ± 0.025
11	30	182	8	0.59 ± 0.019	2604.73 ± 0.035
12	40	133	8	0.47 ± 0.0025	2021.83 ± 0.12
13	30	133	7	0.71 ± 0.003	2401.15 ± 0.14
14	30	133	7	0.70 ± 0.012	2250.30 ± 0.035
15	30	133	7	0.68 ± 0.009	2337.83 ± 0.055

Lipid content was given as area detected into standard experimental conditions

1 $$ \mathrm{Y}={\upbeta}_0+\sum {\upbeta}_{\mathrm{i}}{X}_i+\sum {\upbeta}_{\mathrm{i}\mathrm{i}}{X_i}^2+\sum {\upbeta}_{\mathrm{i}\mathrm{j}}{X}_i{X}_j $$

Where Y is the response variable; β_0_ is a constant; X_i_ and X_j_ are the coded variable ranging between +1 and −1; β_i_, β_ii_ and β_ij_ are the linear, quadratic, and interaction effect coefficients, respectively. The plan of Box-Behnken in coded levels of the independent variables is shown in Tables 1 and 2.

#### Nile red staining of cells

Nile red (NR) (9-(diethyl amino) benzo[a]phenoxazin-5(5H)-one, Sigma–Aldrich) was dissolved in acetone as a stock solution of 250 mg.dm^−3^. After cultivation, microalgae cells were stained with NR (2 μg.cm^−3^) at room temperature, shaken for 1 min on a vortex mixer and incubated for 15 min in darkness. After the incubation period, samples were directly analyzed by flow cytometry (FCM) and optical microscopy.

#### Flow cytometry

Counting and characterization of the selected strain were analyzed by FCM using a LSR flow cytometer (Becton Dickinson Biosciences) equipped with a 488 nm argon laser. The sample cells were entrained in the core of a sheath fluid so that they were individually intercepted by the laser beam, generating scatter and fluorescence signals. The forward scatter signal is related to the cell size and the sideward scatter signal to the cell structure and granularity. In addition to the scatter signals, two fluorescence signals were recorded, namely that of NR orange fluorescence (λExcitation/λEmission; 526/575 nm) after cell-lipid staining, and red fluorescence of chlorophyll *a* (λExcitation/Emission 613/682 nm). The LSR flow cytometer was under the control of the Cell Quest Pro software (Becton Dickinson, UK) that was also used for data recording. It is necessary to note that all the mentioned FCM analyses were run the same day with the same setting of the instrument. The data analysis was conducted with the SUMMIT™ (DAKO) software. Cell clusters were distinguished on the basis of their optical properties.

### Microscopical determination of lipid content in microalgae cells stained by NR

An epifluorescence microscope, Axio Observer Z1 (Carl Zeiss, Oberkochen, Germany), was used to observe at 600 nm the fluorescence of NR-stained microalgae upon excitation at 555 nm, and to take pictures of the stained cells.

### Extraction of total lipids and Gas Chromatography Coupled with Flame Ionization Detector (GC-FID) Analysis

Fatty acid methyl esters (FAME) were produced from microalgal oil according to Laguerre et al. [46]. The analysis of FAME was conducted using gas chromatography (model 6890, Agilent Technologies, SGE, Courtaboeuf, France) equipped with a Supelcowax 10 capillary column (length, 30 m; i.d., 0.32 mm; film thickness, 0.25 mm). Helium was used as a carrier gas with a flow rate of 1 cm^3^. min^−1^. The injector and the Flame Ionization Detection (FID) were kept as 250 °C and 270 °C, respectively. The initial column temperature was set at 150 °C and raised to 225 °C at 5 °C.min^−1^. FAMEs were characterized by Gas Chromatographic (GC) comparison with commercially available FAMEs as internal standards.

### Separation of total lipid content by Thin Layer Chromatography

The separation of polar and neutral lipids was performed by mono-dimensional High Performance-Thin Layer Chromatography (HP-TLC) using Silicagel 60 F254 plates (20 × 10 cm) (Merck, Germany) as a stationary phase [46]. The HP-TLC analysis was carried out for microalgal-oil samples. The mobile phase was composed of chloroforme/methanol/acetic acid (70/30/1; v/v/v) and hexane/ether/acetic acid (95/5/1; v/v/v) for polar lipids (diacylglycerol, monacylglycerol, free fatty acids (FFA), sterols, monogalactosyldiacylglycerol (MGDG), digalactosyldiacylglycerol (DGDG)) and neutral lipids (triacylglycerols (TAG)), respectively. Detection of lipids was performed by spraying the plates with CuSO_4_ solution. Then, the plates were placed in the steriliser for 10 min at 120 °C [46].

## Results and discussion

### Isolation and molecular identification of the microalgae

In the current study, after screening 20 strains isolated from the Mediterranean coast in the region of Sidi Mansour (Sfax, Tunisia), a microalgal species labelled V_2_ was selected for further analysis on the basis of its morphological characteristics, purity, growth rate and oil content in a preliminary study.

To specify the taxonomic position of this microalgal species, the partial 23S region of the DNA gene amplified by PCR was sequenced. Phylogenetic analysis based on 23S rDNA showed that the V_2_ strain was related to a species of the *Tetraselmis* genus (data not shown) with homology percentage of 99%. The phylogenetic position is very near to *Tetraselmis striata.*

### Culture conditions optimization of cell abundance and lipid production by V_2_ strain

Table 2 shows the results of different combinations of three factors (salinity, light intensity and pH) chosen for optimizing V_2_ strain cell abundance and lipid production*.*

Optimal culture conditions and statistical analysis of Box-Behnken Design results were further explored using NemrowdW Software (Table 2).

After identification of factors affecting cell growth and lipid production, the experimental data were fitted by the following Eqs. (2) and (3), second order polynomials established by multiple regression analysis:

2 $$ {\mathrm{Y}}_1=0.697+0.042{X}_2-0.02{X}_3-0.087{X_1}^2-0.092{X_2}^2-0.067{X_3}^2-0.037{X}_1{X}_2+0.028{X}_2{X}_3 $$

3 $$ {\mathrm{Y}}_2=2329.760+469.564{X}_1 + 286.552{X}_2\hbox{--} 642.012{X_1}^2+295.337{X}_1{X}_2 $$

Where Y_1_ is the predicted cell abundance; Y_2_ is the predicted lipid content; X_1_ is salinity; X_2_ is light intensity and X_3_ is pH.

### Analysis of variance (ANOVA) and statistical analysis

ANOVA was used to test the fit significance of quadratic polynomial equation for experimental data. The correlation value was used as a tool to test the quality of the model whereas the p-values were used to check the significance of each coefficient and the interactions between variables. The R^2^ values of 0.982 and 0.985 linked to cell abundance and lipid production according to Eqs. (2) and (3) respectively, indicated a good agreement between the experimental against predicted values for all responses (Tables 3 and 4).

**Table 3:** Variance analysis for cells abundance response

Source of variation	Sum of squares	Degrees of freedom	Mean square	Ratio	Significance (%)	Significance
Regression	0.0962	9	0.0107	30.2537	0.0777^a^	Significant
Residual	0.0018	5	0.0004			
Lack of fit	0.0013	3	0.0004	1.8571	36.9	Not significant
Error	0.0005	2	0.0002			
Total	0.0980	14				

R^2^ = 0.982, ^a^Significant at 99.9%

**Table 4:** Variance analysis for lipid production response

Source of variation	Sum of squares	Degrees of freedom	Mean square	Ratio	Significance (%)	Significance
Regression	4.45871 E + 0006	9	4.95413E + 0005	35.8982	0.0514^a^	Significant
Residual	6.90025 E + 0004	5	1.38001 E + 0004			
Lack of fit	5.75269 E + 0004	3	1.92034 E + 0004	3.3420	23.9	Not significant
Error	1.14755 E + 0004	2	5.73777 E + 0003			
Total	4.52772 E + 0006	14				

R^2^ = 0.985, ^a^Significant at 99.9%

Furthermore, the analysis of validity values indicated that the lack of fit of model terms was not significant (*P* > 0.05) which was considered a cue for the good quality of both models (Tables 3 and 4).

**Fig. 1:**
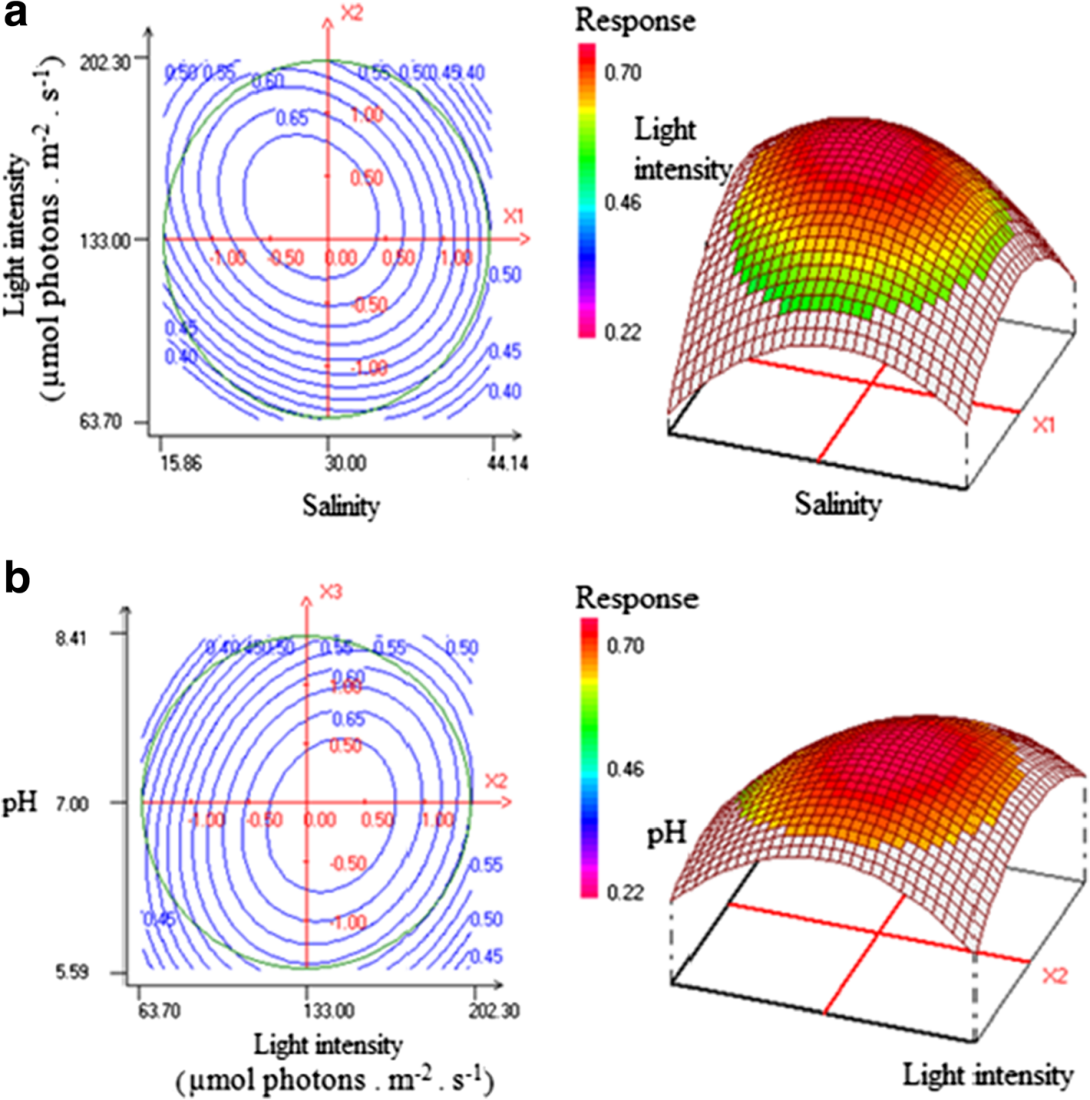
Contour plots and the corresponding cells abundance response surface plot. **a** Effects of salinity and light intensity on cell abundance. **b** Effects of pH and light intensity on cell abundance

The ANOVA analysis revealed that the quadratic effect of both, salinity (X_1_^2^) and light intensity (X_2_^2^), on cell abundance was significant (*P* < 0.05) (Table 5), which was confirmed by the two (2D) and three (3D) dimensional response surface plots displayed in Fig. 1.

**Table 5:** Coefficient statistical-analysis for cell abundance response

Coefficients	Coefficient values	Student test	Significance (%)
b0	0.697	64.19	<0.01^a^
b1	−0.015	−2.26	7.4
b2	0.042	6.40	0.139^b^
b3	−0.020	−3.01	2.98^c^
b11	−0.087	−8.90	0.029^a^
b22	−0.092	−9.41	0.022^a^
b33	−0.067	−6.86	0.101^b^
b12	−0.037	−3.99	1.04^c^
b13	−0.023	−2.39	6.2
b23	0.028	2.93	3.28^c^

^a^Significant at 99.9%, ^b^Significant at 99%, ^c^Significant at 95%

A cross referencing to ANOVA analysis confirmed that the salinity (X_1_), light intensity (X_2_), quadratic salinity effect (X_1_^2^) and interaction between both factors (X_1_X_2_) were actually very significant (*P* < 0.05) in promoting lipid production by V_2_ strain cells grown in F/2 medium (Table 6).

**Table 6:** Coefficient statistical analysis for lipid production response

Coefficients	Coefficient values	Student test	Significance (%)
b0	2329.760	34.35	<0.01^a^
b1	469.564	11.31	<0.01^a^
b2	286.552	6.90	0.0980^a^
b3	89.784	2.16	8.3
b11	−642.012	−10.50	0.0136^a^
b22	−156.325	−2.56	5.1
b33	−48.402	−0.79	46.4
b12	295.337	5.03	0.401^b^
b13	−76.625	−1.30	24.9
b23	95.357	1.62	16.5

^a^Significant at 99.9%, ^b^Significant at 99%

### Culture condition effects on V_2_ strain growth

According to Khatoon et al. [47], it was demonstrated that salinity, light intensity and pH were the important factors that influence the green-microalgae growth. Considering the contour plots and the corresponding 3D response surface plot (Fig. 1), the thighest cell abundance (A_680nm_ = 0.7 ± 0.01) was observed at intermediate values for pH (7), salinity (30) and light intensity (133 μmol photons.m^−2^.s^−1^) (Fig. 1a and b). Therefore, the growth of this strain was reduced at low and at high salinity because of accumulation of osmo-protectant solutes shielding enzyme metabolism [48]. Similarly, Bartley et al. [49] reported that high salinity resulted in cell-abundance decrease, which was also confirmed by the study of Khatoon et al. [47] when *Nannochloropsis* sp. and *Tetraselmis* sp. were cultured at salinity: 40. These results were further supported by Allakhverdiev et al. [50] who reported that the high salinity inhibited protein synthesis by inactivating ATP-synthase.

For light intensity stress, Cheirsilp and Torpee [51] showed that the maximum level of *Nannochloropsis* sp. growth was obtained by increasing light intensity up to 10 000 Lux (135 μmol photons.m^−2^.s^−1^) which was in accordance with findings of this study (133 μmol photons.m^−2^.s^−1^). Chen et al. [52] stated also that the high light intensity inhibited the growth of microalgae which was consistent with the absorbance decrease observed when *Tetraselmis* sp. was cultivated under high light intensity. Khatoon et al. [47] reported that the highest absorbance of *Nannochloropsis* sp. cultures was reached at pH 8.5. The above reported experiments are also supported by findings of Khalil et al. [53] who reported that the *Dunaliella bardawil* dry-mass production was maximal at pH 7.5 and lower at pH 10 and pH 4.

### Culture condition effects on lipid content in V_2_ strain

Any fluctuation of several factors lead to the production of intracellular lipids. It was reported by Mata et al. [29] and Khatoon et al. [47] that *Tetraselmis* sp. and *Nannochloropsis* sp. were the best known microalgae that produce high lipid content (20–50% DW).

**Fig. 2:**
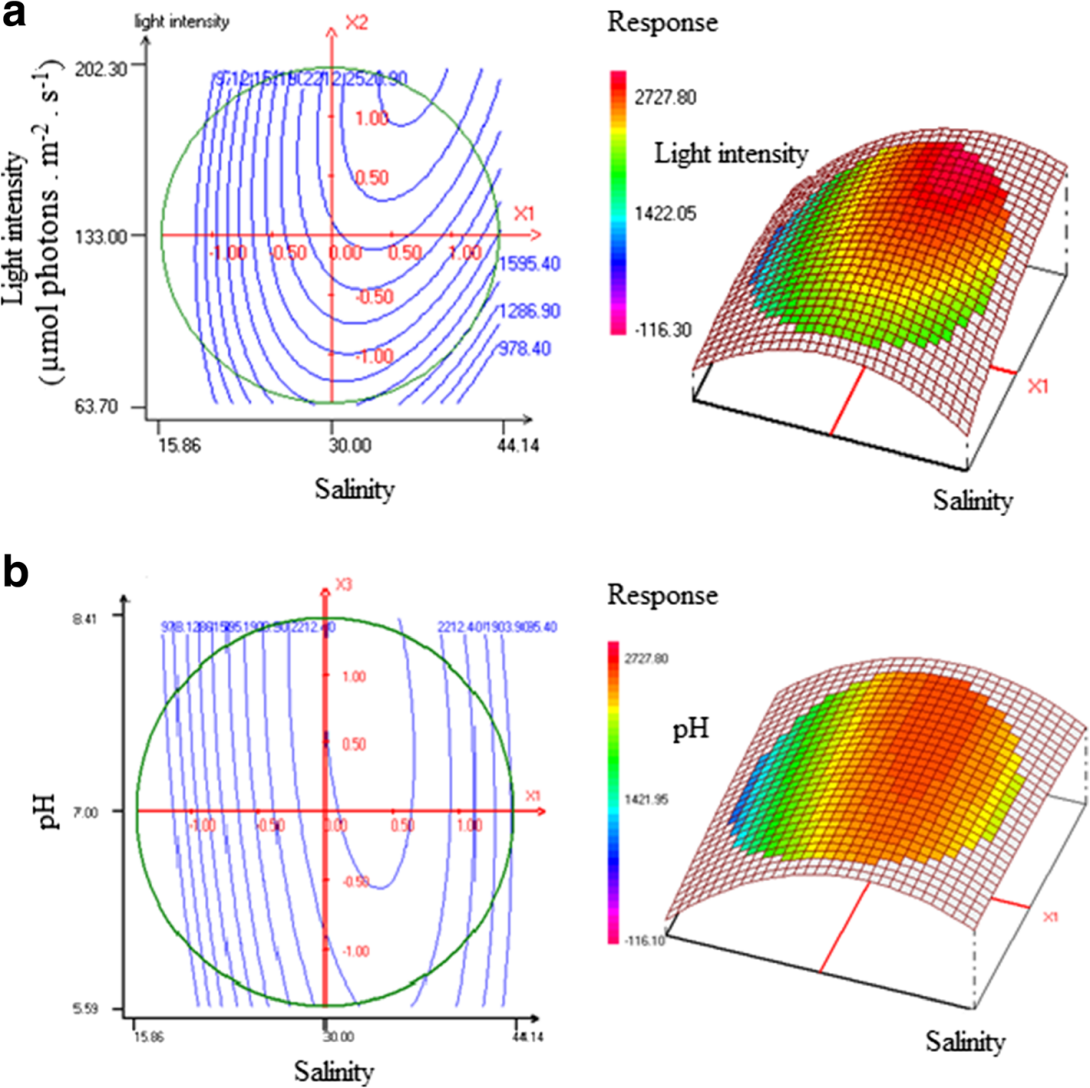
Contour plots and the corresponding lipid response surface plot. **a** Effects of salinity and light intensity on lipid production. **b** Effects of pH and light intensity on lipid production

To investigate the interaction between culture conditions (salinity, light intensity and pH) on V_2_ strain lipid production*,* the two and three dimensional response surfaces were considered (Fig. 2).

When maintaining the light intensity at 133 μmol photons.m^−2^.s^−1^, the lipid production increased gradually with pH increasing from 6 to 7.6 and salinity ranging from 20 to 33.6 (Fig. 2b). This result disagreed with observations by Dahmen et al. [40] who showed that an increase in salinity inhibited cell growth as well as lipid synthesis of *Picochlorum* sp. Considering the cell abundance results, the best lipid production was achieved when light intensity was at its maximum level (188 μmol photons.m^−2^.s^−1^), the salinity at 37.23 and pH at 7 (Fig. 2a). On the other hand, the effect of pH (X_3_) on lipid production was not significant (Table 6).

Similar investigations have been performed by other researchers using alternative microalgae species. The investigations conducted with *Scenedesmus abundans* showed that the highest lipid content was 32.77% DW under a light intensity of 6000 Lux (resembling a PAR of 81 μmol photons.m^−2^.s^−1^ when using our lamps), whereas, the lowest lipid content (21.20% DW) corresponded to a light intensity of 3000 Lux (40.5 μmol photons.m^−2^.s^−1^) [20]. Independtly*, Nannochloropsis oleoabundans* HK-129 produced its highest lipid content under a light intensity of 14 800 Lux (199.8 μmol photons.m^−2^.s^−1^) [15]. According to He et al. [10], the maximum lipid content of *Chlorella* sp. L1 (33.03% DW) was reached under a light intensity of 400 μmol photons.m^−2^.s^−1^.

Any salinity variation would induce biochemical and physico-chemical changes in microalgae. This statement was supported by Kalita et al. [54] who reported that salinity stress resulted in an increased lipid production that was related to changes in fatty acid metabolism. According to Hu [55], a salinity increase from 10 to 35 would lead to a lipid content increase in microalgae. With the highest lipid content observed at a salinity of 37.23, the present study is in agreement with these findings. Talebi et al. [56] demonstrated that the osmotic pressure on microalgae cells activated several responses such as regulation of ion transport through the plasma membrane, and synthesis of stress proteins to maintain a constant growth and to accumulate osmo-protectant solutes. These processes were paralleled by an increased lipid accumulation. Similarly to the results reported by Khatoon et al. [47], the maximum lipid content produced in the present study was reached at pH 7. According to Bondioli et al. [57], the lipid content produced by *Tetraselmis suecica F&M-M33* amounted to 22, 27 and 29% DW under nitrogen starvation, nitrogen and phosphorus starvation, and nutrient repletion, respectively.

**Fig. 3:**
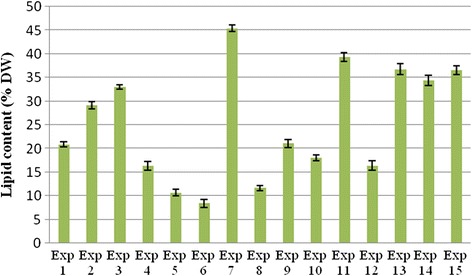
*Tetraselmis* sp. lipid content (% DW) determined by the gravimetric method under different culture conditions

The main novelty of the present work was to investigate the combination and interaction effect of three important environmental factors (pH, salinity and light intensity). This way, growth conditions could be identified, which lead to the highest lipid production level (49% DW) by V_2_ strain. This is a lipid content higher than that reported by Khatoon et al. [47] as well as other studies. The highest *Tetraselmis* sp. lipid content determined by gravimetric method corresponded to culture condition 7 (45.4 ± 0.2% DW), followed by culture condition 11 (39.31 ± 0.013% DW) (Fig. 3). These values were higher than that reported by Hu et al. [58] for oleaginous green algae. *Tetraselmis* sp*.* was also singled out as a high lipid producer in previous studies [22, 59].

### Validation of the model

The culture conditions optimized for cell abundance were as follows: salinity: 30; light intensity: 133 μmol photons.m^−2^.s^−1^; pH: 7. The optimized culture conditions for lipid production were salinity: 37.23; light intensity: 188 μmol photons.m^−2^.s^−1^; pH: 7.

After 15 days of V_2_ strain growth under optimized culture conditions, cell absorbance and lipid production reached 0.7 ± 0.01 and 49 ± 2.1% DW, respectively. The lipid production was 2.6 times higher than that obtainable with cultures in F/2 medium under standard conditions: salinity: 41; light intensity: 84 μmol photons.m^−2^.s^−1^ and pH: 7.6, which emphasizes the *Tetraselmis* sp. high efficiency in lipid production. These results demonstrate the great usefulness of the Bok-Behnken methodology to optimize growth factors and study their interaction in order to achieve the highest oil production.

### Flow cytometry and epifluorescence microscopy observations

**Fig. 4:**
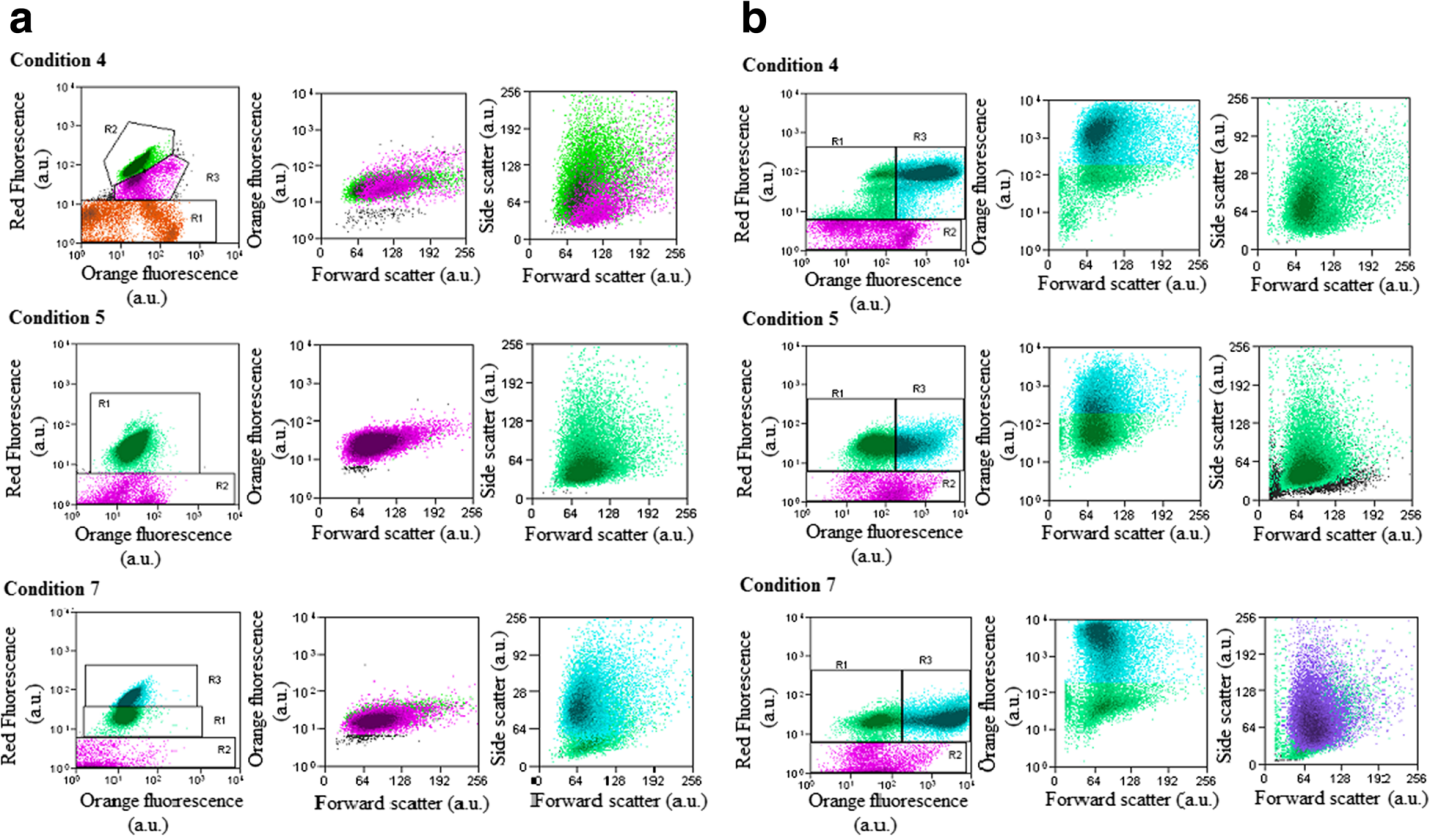
Cytograms of *Tetraselmis* sp. cells under various culture conditions (4, 5 and 7). **a** Unstained *Tetraselmis* sp. cells under culture conditions (4, 5 and 7). **b** Nile red stained *Tetraselmis* sp. cells under culture conditions (4, 5 and 7)

In stressed culture, neutral lipids (NL) are the major component of microalgal oils. These NL represent total lipids stored in cells. They consist in energy reserve bodies of TAG [60]. The microalgae lipid content was quantified by FCM after staining the intracellular lipid bodies with the lipophilic dye NR [61, 62], as reported by Teo et al. [63]. Silva et al. [64] established a linear correlation between the total NR fluorescence recorded by FCM and the total microalgal lipid content determined by the gravimetric method [65, 66], making NR staining a promising method for cell lipid quantification. Figure 4 displays typical FCM cytograms of V_2_ strain grown under conditions 4, 5 and 7.

The dot plots representing red fluorescence versus orange fluorescence or orange fluorescence versus the forward scatter signal reveal the existence of two subgroups differentiated by their fluorescence intensities. Two subgroups were observed under condition 4 (Fig. 4a). Each cluster is characterized by the median values of its variables that are reported in Table 7. In particular, V_2_ strain under condition 4 shows the largest median of red fluorescence, as well as the largest median of the size value. This is suggesting a “healthier” (larger chlorophyll content, larger size) cell production under condition 4 than under conditions 5 and 7.

**Table 7:** Median values of *Tetraselmis* sp. flow cytometric variables

Conditions (4- 5–7)	Red fluorescence (a.u.)	Size (a.u.)	Structure (a.u.)	Orange fluorescence (a.u.)
Unstained cells				
Condition 4	85	97	80	24.01
Condition 4-R2	91.37			24.01
Condition 4-R3	37.04			27.74
Condition 5	27.74	89	53	24.89
Condition 7	42.79	82	88	16.4
Condition 7-R1	22.34			15.01
Condition 7-R3	57.13			19.33
Nile red stained cells				
Condition 4	73.56	90	79	481
Condition 4-R1	34.46			81.98
Condition 4-R3	85			1230.82
Condition 5	24.89	82	54	557.69
Condition 5-R1	26.76			44.75
Condition 5-R3	27.74			811.97
Condition 7	20.78	81	68	536.31
Condition 7-R1	20.04			49.44
Condition 7-R3	24.01			2724.55

Median values of *Tetraselmis* sp. flow cytometric variables were obtained in absence and presence of NR stained cells.

The cytograms corresponding to the NR stained samples (Fig. 4b) prove that not all *Tetraselmis* sp. cells respond to NR. Under condition 7, the subgroup with the highest median red fluorescence gave rise to the largest response to NR. Indeed, its median orange fluorescence was 2-fold more than that of the responding cells under condition 4 and 3-fold more than that of cells under condition 5, in agreement with results reported in Table 2. Thus, the increase in cell lipid content appears to be linked to the light intensity increase from 84 to 182 μmol photons.m^−2^.s^−1^. This is consistent with the fact that low irradiance reduces CO_2_ assimilation as reported by Beardall et al. [67].

These experiments demonstrate that cell growth and lipid production are not tightly coupled and that the choice of the growth conditions depends on the selected priority, either biomass or lipids. The fact that a subpopulation of *Tetraselmis* sp. does not respond to NR staining deserves further investigation. Indeed, it would be important to find out if the observed cluster heterogeneity expresses real species heterogeneity. If so, the subspecies exhibiting the highest lipid content should be characterized to obtain the optimal lipid production. This subspecies seems to correspond to the one with the highest chlorophyll content (Fig. 4b) which excludes the possibility to link its existence to an artifact of NR staining. Consequently, the FCM quantification open a way to further optimize lipid production by V_2_ strain.

**Fig. 5:**
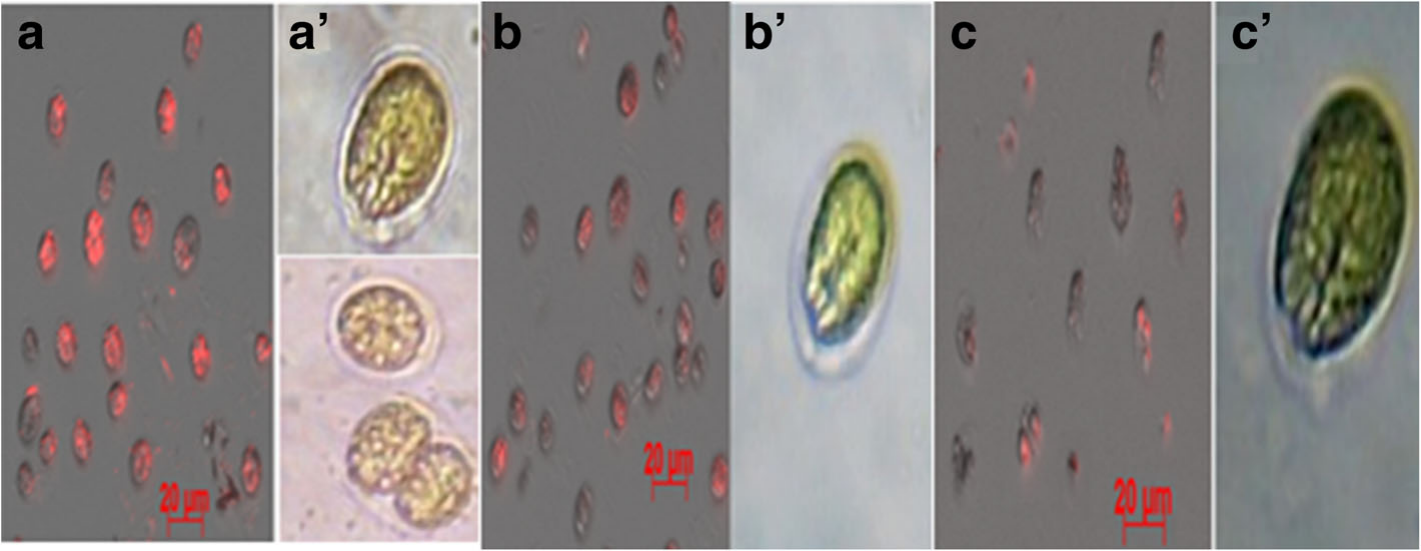
Fluorescence microscopy images of *Tetraselmis* sp. grown under different conditions. (**a**, **b** and **c**) correspond to fluorescence images of cells stained with NR (Excitation wavelength centered at 555 nm and fluorescence emission wavelength centered at 600 nm) to highlight the lipid droplets (red) cultured under indicated conditions. (a’, b’ and c’) correspond to phase contrast images of corresponding culture conditions. (**a** and a’) culture condition 7. (**b** and b’) culture condition 4. (**c** and c’) culture condition 5. All images of epifluorescence microscopy were taken at × 20 magnification, while contrast images were taken at × 40 magnification. Scale bar represents 20 μm

The presence of lipid droplets inside *Tetraselmis* sp. cells revealed by NR staining was also observed by epifluorescence microscopy (Fig. 5). The photomicrographs showed the highest lipid accumulation inside cells cultured under condition 7, followed by cells cultured under condition 4, whereas the lipid content was very low under condition 5 (Fig. 5). These observations are fully consistent with those made by FCM.

### Fatty acids composition

Similar to plants, many species of microalgae can accumulate polar and neutral lipids as energy and carbon storage. In this study, results obtained with HP-TLC revealed that TAG, 1,2 diacylglycerol, 1,3 diacylglycerol and FFA were the principal components of *Tetraselmis* sp. extracted lipids. The *Tetraselmis* sp. fatty acid composition as determined by GC-FID analysis is displayed in Table 8.

**Table 8:** Fatty acids composition and total lipid content of *Tetraselmis* sp.

Name	% of the total FAME
Saturated fatty acids	
Myristic (14:0)	0.82
Palmitic (16:0)	30.89
Heptadecanoic (17:0)	2.99
Stearic (18:0)	1.07
(19:0)	7.25
Arachidic (20:0)	1.62
Total saturates	44.68
Monounsaturated fatty acids	
Palmitoleic (16:1)	5.58
Oleic (18:1)	32.88
Gadoleic (20:1)	0.73
Total monounsaturates	39.20
Polyunsaturated fatty acids	
Linoleic (18:2)	8.12
Linolenic (18:3)	1.84
Dihomolinoléic (20:2)	1.85
DGL (20:3)	0.85
EPA (20:5)	3.43
Total polyunsaturates	16.10
Total lipids (% DW)	49 ± 2.1

Percentages values of fatty acids and total lipid content were measured when *Tetraselmis* sp. was cultured under optimized culture conditions for lipid production. The chemical formula of each fatty acid is indicated in parenthesis.

Harwood [68] reported that light intensity fluctuation can change the metabolism of lipids in green microalgae with modification of lipid class composition. Therefore, light intensity variations can modulate fatty acid synthesis.

In this study, V_2_ strain reached a total lipid content of 49 ± 2.1%, a value higher than the total lipid content found in *Picochlorum* sp. SBL2 (25.28 ± 2.38%), *Nannochloris* sp. SBL1 (19.69 ± 2.19%), *Nannochloris* sp. SBL4 (22.65 ± 2.21%) and *Desmochloris* sp. SBL3 (23.16 ± 3%) by Pereira et al. [69]. Hu et al. [58] reported that oleaginous chlorophyte can accumulate lipids up to an average of 25% DW. GC profile showed a dominance of saturated and mono-saturated fatty acids representing more than 80% of the total fatty acid methyl esters (FAMEs). Oleic (C18:1) and Palmitic (C16:0) acids were the predominant fatty acids in autotrophic microalgae cultures, accounting for 32.88% and 30.89% of the total FAMEs, respectively. The finding that the third most abundant fatty acid, linoleic acid (C18:2) accounted for 8.12% of total fatty acids is in good agreement with reported contents in *Monoraphidium* [26], *Nannochloris. SBL1* and *Nannochloris* sp. [69]. V_2_ strain was found to contain an important proportion of PUFAs and MUFAs, which accounted for 16.1% and 39.2% of the total FAMEs, respectively. These values were similar to results obtained by Selvakumar and Umadevi [70] who reported that the highest level of PUFAs (17.7%) in *T. gracilis* was observed at low concentration of nitrate (0.05 g.dm^−3^). Moreover, the fatty acid profile showed the presence of important levels of *omega 3* (*ω3*), *omega 6* (*ω6*) and *omega 9* (*ω9*) that reached 5.28, 8.12 and 32.8% of the total FAMEs, respectively. The EPA percentage amounted for 21.3% of total PUFAs, while a lower percentage of this fatty acid (14% of total PUFAs) was recorded at 0.05 g.dm^−3^ of nitrate in *T. gracilis* [70].

## Conclusions

The oleaginous microalga *Tetraselmis* sp. was isolated and identified on the basis of 23S rRNA gene. Optimization of light intensity, salinity and pH were crucial factors for oil production by green microalgae. In this work, a high cell density (A_680nm_, 0.7) was achieved by the V_2_ strain, when cultured at the following conditions: salinity: 30, photosynthetic active light intensity: 133 μmol photons.m^−2^.s^−1^, and pH: 7. The highest lipid content (49% DW) was reached under the following conditions: salinity: 37.23; photosynthetic active light intensity: 188 μmol photons.m^−2^.s^−1^, and pH: 7. In the experiments presented here, it was demonstrated that combining several culture conditions can significantly enhance lipid production by this strain. In *Tetraselmis* sp., the percentage of total PUFAs was 16.1% under optimized culture conditions for lipid production. Results show the possibility of increasing growth, total lipid and fatty acid contents (essentially PUFAs), in *Tetraselmis* sp. cultures. The isolated *Tetraselmis* sp. strain proved to be ideal for lipid and PUFAs production.

## Abbreviations

2D: Two dimensions
3D: Three dimensions
A: Absorbance
a.u.: Arbitrary unit
ANOVA: Analysis of variance
BBD: Box-Behnken design
DGDG: Digalactosyldiacylglycerol
DW: Dry weight
EXP: Experiment
FAME: Fatty acid methyl ester
FCM: Flow cytometry
FFA: Free fatty acids
FID: Flame ionization detection
GC: Gas chromatography
HP-TLC: High performance-thin layer chromatography
MGDG: Monogalactosyldiacylglycerol
MUFA: Mono-saturated fatty acids
NL: Neutral lipids
NR: Nile red
PUFAs: Poly-unsaturated fatty acids
R: Subgroup
RSM: Response surface methodology
TAG: Triacylglycerols
*ω*3: *Omega* 3
*ω*6: *Omega* 6
*ω*9: *Omega* 9
